# White Matter Changes and Word Finding Failures with Increasing Age

**DOI:** 10.1371/journal.pone.0014496

**Published:** 2011-01-07

**Authors:** Emmanuel A. Stamatakis, Meredith A. Shafto, Guy Williams, Phyllis Tam, Lorraine K. Tyler

**Affiliations:** 1 Division of Anaesthesia, School of Clinical Medicine, University of Cambridge, Cambridge, United Kingdom; 2 Centre for Speech, Language and the Brain, Department of Experimental Psychology, University of Cambridge, Cambridge, United Kingdom; 3 Wolfson Brain Imaging Centre and Department of Neurosurgery, University of Cambridge, Cambridge, United Kingdom; University of Granada, Spain

## Abstract

**Background:**

Increasing life expectancy necessitates the better understanding of the neurophysiological underpinnings of age-related cognitive changes. The majority of research examining structural-cognitive relationships in aging focuses on the role of age-related changes to grey matter integrity. In the current study, we examined the relationship between age-related changes in white matter and language production. More specifically, we concentrated on word-finding failures, which increase with age.

**Methodology/Principal Findings:**

We used Diffusion tensor MRI (a technique used to image, *in vivo*, the diffusion of water molecules in brain tissue) to relate white matter integrity to measures of successful and unsuccessful picture naming. Diffusion tensor images were used to calculate Fractional Anisotropy (FA) images. FA is considered to be a measure of white matter organization/integrity. FA images were related to measures of successful picture naming and to word finding failures using voxel-based linear regression analyses. Successful naming rates correlated positively with white matter integrity across a broad range of regions implicated in language production. However, word finding failure rates correlated negatively with a more restricted region in the posterior aspect of superior longitudinal fasciculus.

**Conclusions/Significance:**

The use of DTI-MRI provides evidence for the relationship between age-related white matter changes in specific language regions and word finding failures in old age.

## Introduction

With life expectancy increasing substantially it is becoming progressively more important to understand the neurophysiological underpinnings of age-related cognitive changes. Imaging techniques such as Magnetic Resonance Imaging (MRI) enable us to study age-related physiological changes *in vivo* in parallel with age-related cognitive changes, thus establishing relationships between physiology and cognition.

The majority of studies examining the relationship between age-related changes in brain structure and cognition focus on grey matter (GM). Early studies adopted practices from neuropsychology and used manual outlining of brain structures to assess changes across the lifespan and their impact on cognition [1]. Although this type of approach is still popular, the use of automated methods that require minimal operator involvement is now the norm. Volumetric studies have thus become more reliable in producing robust observer independent findings [2]–[4]. Volumetric findings on age-related structural changes in white matter diverge in that some studies report widespread white matter changes with age [5] while other studies suggest that global white matter does not decline with age [2]. White matter comprises bundles of myelin-coated axons (tracts) that conduct neural information between GM regions (effectively connecting GM regions) so its role is crucial if the brain is to function in a coordinated manner.

A technique that has gained remarkable popularity in the past ten years as means for studying white matter integrity is Diffusion Tensor MRI (DTI), used to image, *in vivo*, the diffusion of water molecules in brain tissue. Diffusion weighted images are used to calculate tensors providing information on local white matter fiber tract direction fields, and regional white matter tissue composition is expressed by measures such as Fractional Anisotropy (FA); [6]. FA in white matter originates roughly from the presence and coherence of oriented structures and higher FA values have been related to increases in white matter integrity [7]. Large FA values suggest highly restricted diffusion and consequently highly organized myelinated structures. FA images are more sensitive to differences in white matter microstructure than T1-weighted images [8] so using voxel based assessment of FA images offers advantages over volumetric assessment of T1 weighted MRI images. Moreover, in a comparison between DTI and magnetization transfer imaging (MTI), DTI measures were shown to be remarkably sensitive to age-related white matter changes in that DTI parameters, and particularly FA, correlated more strongly than MTI with both age and scores in various cognitive tests assessing working memory, executive function and episodic memory [9].

Studies linking WM measures with cognition [10] in aging have tended to focus on frontal lobe functions (e.g. attention, decision making) and prominent white matter structures such as the corpus callosum, which are readily identified and measured. The neural underpinnings of other types of age-related cognitive decline, such as language, have enjoyed less attention despite the fact that language difficulties such as word-finding failures constitute a major complaint as we age [11].

Word-finding difficulties are exemplified in the tip of the tongue states (TOTs) where people are temporarily unable to produce a word they are certain they know. TOT frequency increases with normal aging and behavioral evidence suggests that the underlying problem is in retrieving the complete phonology of the target word during production [12], [13]. The implication of these findings is that healthy aging may affect fundamental processes of word retrieval and language production.

Successful naming involves multiple processes potentially requiring the coordination of wide-ranging perisylvian brain regions primarily involving superior and middle temporal gyri and inferior frontal gyri in the left hemisphere [14]. Our previous investigations into age-related grey matter atrophy and word retrieval processes have revealed that atrophy in the left insula, an area implicated in phonological production, contributes to increased TOTs [15].

Having already established the contribution of grey matter changes to age-related word finding difficulties [15], in the present study we investigate the role of age-related changes in the white matter connections which are essential for the overall integrity of the distributed neural networks involved in language, and other cognitive functions. There has been an increasing number of studies in the past few years emphasizing the importance of fronto-temporal white matter bundles such as the arcuate and inferior longitudinal fasciculi [16], [17] and extreme capsule [18], [19] in language function. While these studies use tractography, a technique that utilizes DTI data to generate three dimensional computerized representations of specific white matter fiber pathways, in the present study we carry out a detailed voxel based assessment of the effect of aging on white matter tracts throughout the brain utilizing FA. We also aimed to determine whether age-related changes affect word retrieval processes by correlating rates of successful and failed retrieval with white matter integrity (as measured by FA) in subjects across a wide age range. More specifically, we asked whether age-related changes in white matter integrity are associated with age-related increases in word-finding difficulties as measured by TOT rates, and whether changes to white matter integrity also impact more broadly on word retrieval processes.

We hypothesize that the success of the functional networks active during word production depends on the integrity of connective white matter tracts, and that these tracts are susceptible to age-related deterioration. While successful retrieval may be related to white matter integrity throughout the production network we expect TOT rates to be related to changes only in specific regions that reflect the locus of retrieval failure, namely phonological retrieval, such as the superior longitudinal fasciculus (SLF) [20].

## Methods

### 1. Participants

This research was approved by the Cambridgeshire Research Ethics Committee. All participants gave informed written consent before participating in the study. Participants were 28 healthy adults 19 to 82 years old (*M* = 52.4, *SD* = 18.46) recruited from the University of Cambridge and surrounding community. The 28 participants were a subset of a larger group of volunteers that took part in a volumetric study in which we related word finding failures to age-related grey matter declines [15]. All volunteers had normal or corrected to normal hearing and vision during behavioral testing. All volunteers scored 26 or above on the MMSE [21] (*M* = 29.3, *SD* = 1.13), and scores did not correlate significantly with age (*p*>.1). Major exclusion criteria for participation included MR contraindications, neurological or hormonal disorders, recent treatment (within one year) for psychiatric disorders, major head trauma, stroke, dyslexia (self-report), bilingualism (self-report), and left-handedness (self-report or less than 50% right-handed on the Edinburgh Handedness Inventory; [22].

### 2. Behavioral Tasks

Volunteers completed 2–3 behavioral sessions each lasting 60 to 90 minutes. Testing included a number of background measures, to assess current and past health status and to identify potential exclusion criteria. Additionally we used the Edinburgh Handedness Inventory [22] to confirm self reported handedness, and administered an audiometer test to examine hearing. Cognitive and language abilities were assessed with the Mini Mental State Exam (MMSE); [20], a screening test for dementia, and the National Adult Reading Test (NART); [23], a measure of verbal IQ which involves pronouncing 50 irregularly-spelled words. Volunteers were asked to complete measures of digit span forward and backward [24], a standardized 40-item vocabulary test [25], and the Boston Naming Test (BNT); [26], which involves naming 60 simple line drawings. These background measures provided data on volunteers' cognitive abilities including verbal IQ, working memory, vocabulary, picture naming.

**Figure 1:**
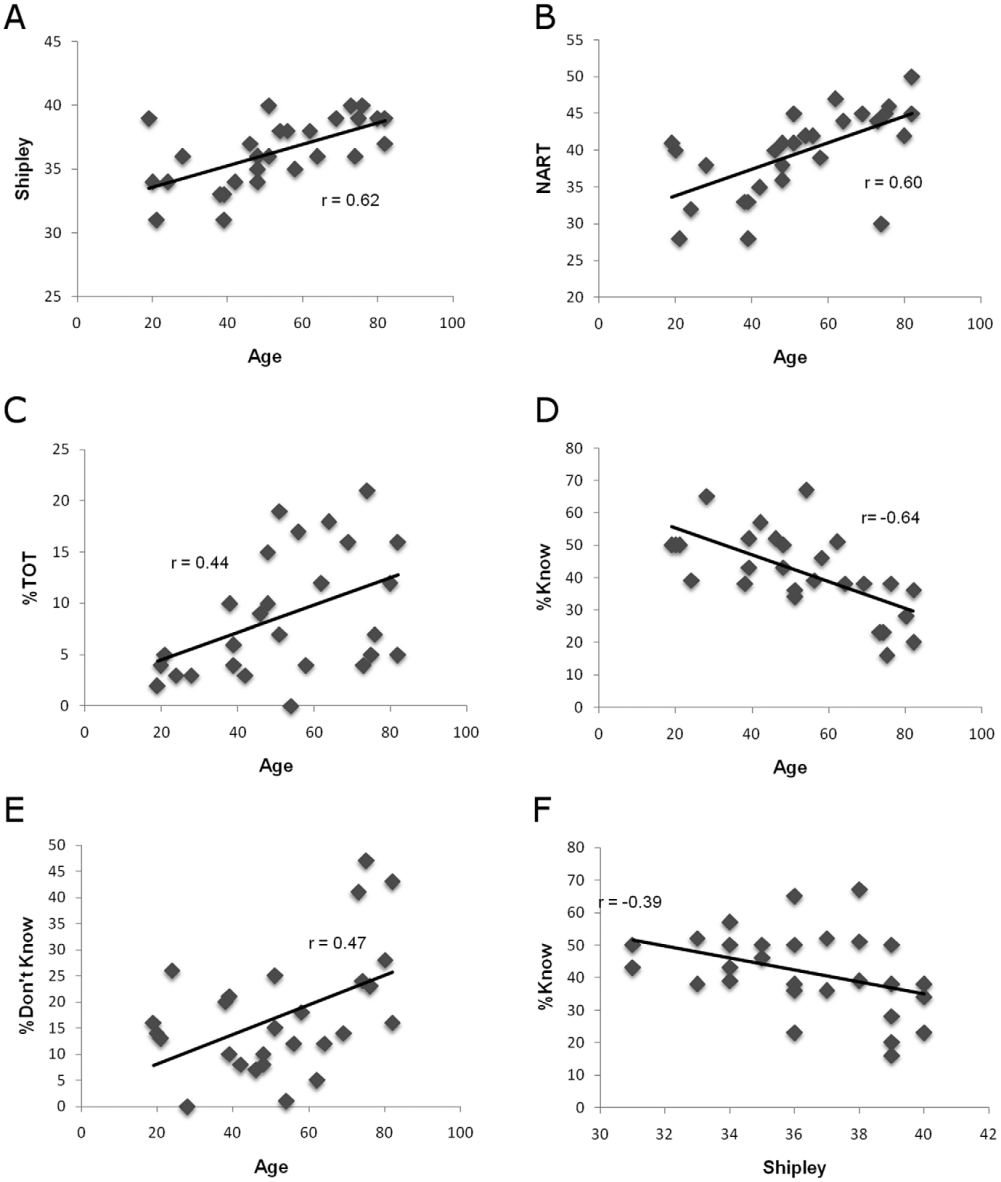
Relationships between background measures, scores from the TOT task and age. The plots show the relationship between: a) age and Shipley scores (*r* = .62, *p*<.001), b) age and NART scores (*r* = .60, *p*<.001), c) age and TOT rates (*r* = .44, *p*<.01), d) age and Know rates (*r* = −.64, *p*<.001), e) age and Don't know rates (*r* = .47, *p*<.05) and f) Know rates and Shipley scores (*r* = −.39, *p*<.05).

Our experimental task of interest was the TOT task for which we employed a procedure similar to previous studies [15]. Sixty-eight pictures of public figures from different occupational categories (e.g., movie stars, politicians, sports figures, authors, etc.) were presented in a single randomized order, with a short written description underneath each picture. The stimuli were selected from a larger set on the basis of familiarity and ability to induce TOTs in pilot testing with 10 young and older adults. Volunteers were asked to name the person and were instructed to respond with the name of the person (Know), to say they did not know the name (Don't Know), or to say they were having a TOT for the name (TOT). Responses were not timed, but if participants were unresponsive for several seconds, the experimenter prompted them for an answer choice. When TOTs occurred volunteers were encouraged to provide any partial information about the name (e.g., initial syllable or length) or alternative names that were coming to mind. Following each TOT trial, volunteers were given the correct target name and asked to confirm that they were having a TOT for the target name. Plots of some of the background measures and scores from the TOT task are shown plotted against age in Figure 1.

### 3. DTI data Acquisition

All scans were carried out at the Wolfson Brain Imaging Centre, Cambridge using a Bruker MedSpec S300 3T scanner. A 63 directions encoding scheme with a b-value of 1000 s/m^2^ was used. The encoding scheme follows the directions of Hardin [27] which come from repulsion simulations of charges bound to the surface of a sphere. Diffusion weighting was achieved using a Stejskal-Tanner sequence with pulse width δ = 27.5 ms and inter-pulse spacing Δ = 40 ms [28]. Four b = 0 s/mm^2^ images were also acquired. The total DTI scan time was 12 minutes. We used the DTI data to calculate Fractional Anisotropy (FA) images, a rotationally invariant measure of the directionality of the diffusion in a voxel [29] and we report analyses of the FA images. An isotropic medium will have zero fractional anisotropy, while a highly anisotropic voxel will have FA values close to unity. FA values have been related to increases in white matter organization/integrity [7].

### 4. DTI data analysis

#### Preprocessing

The FA images were co-registered to reduce the effect of eddy currents and volunteer movement using in-house software following an algorithm described by Andersson and Skare, 2002 [30]. The base EPI images were spatially normalized with linear and nonlinear components (12 affine, cut-off used for DCT bases functions  = 25) to the SPM EPI template (Wellcome Department of Cognitive Neurology, London, UK). The linear and non-linear normalization parameters were then applied to the raw FA images that were in native space up to this point. Visual inspection revealed that the spatial normalization process did completely succeed in bringing the FA images into standard space. We rated how well the images were normalized and established the best normalized FA image. This ‘best normalized’ image acted as a template to which all other FA images were coregistered using linear affine transformations only. An average of all the coregistered images and their flipped (along the *x*-axis) versions was obtained and this acted as a symmetrical FA template. We thus constructed a template in the same modality as the images we wanted to normalize and we spatially normalized raw FA images (with linear affine and nonlinear parameters) to this modality-specific template. We created a symmetrical template in order to allow the comparison of anisotropy between left (LH) and right (RH) hemispheres [31]. Visual inspection revealed this optimized spatial normalization technique produced satisfactory spatial normalization for the FA image of every volunteer.

The spatially normalized FA images were smoothed with a 10 mm isotropic Gaussian filter and were statistically modeled using the General Linear Model in SPM5. Linear regression analyses were used to identify clusters with a significant correlation between FA values and age, or between FA and behavioral scores (e.g., proportion of TOTs) across volunteers. We thresholded the statistical parametric maps at *p*<.001 (unless otherwise specified), uncorrected at the voxel level and report maxima for clusters that survive a random field corrected *p*<.05, corrected for white matter volume across the entire brain. The correction for the entire white matter volume was implemented using a white matter mask from the WFU Pick Atlas [32]. Besides age (used to assess age related white matter integrity changes), we correlated FA with laterality (to assess differences in white matter integrity when comparing the two hemispheres), Know rates (to assess which white matter tracts are important for successful word retrieval) and finally TOT rates (to assess whether age related deterioration in integrity in specific tracts affects word retrieval).

We also attempted to establish whether part of the FA variance can be uniquely explained by proportion TOTs once the FA variance attributable to aging has been accounted for. To investigate this we carried out a multiple linear regression using both TOT scores and age as predictor variables in the same model. In this manner we calculated the independent contribution of each of these variables (age and TOT scores) to FA variance.

**Figure 2:**
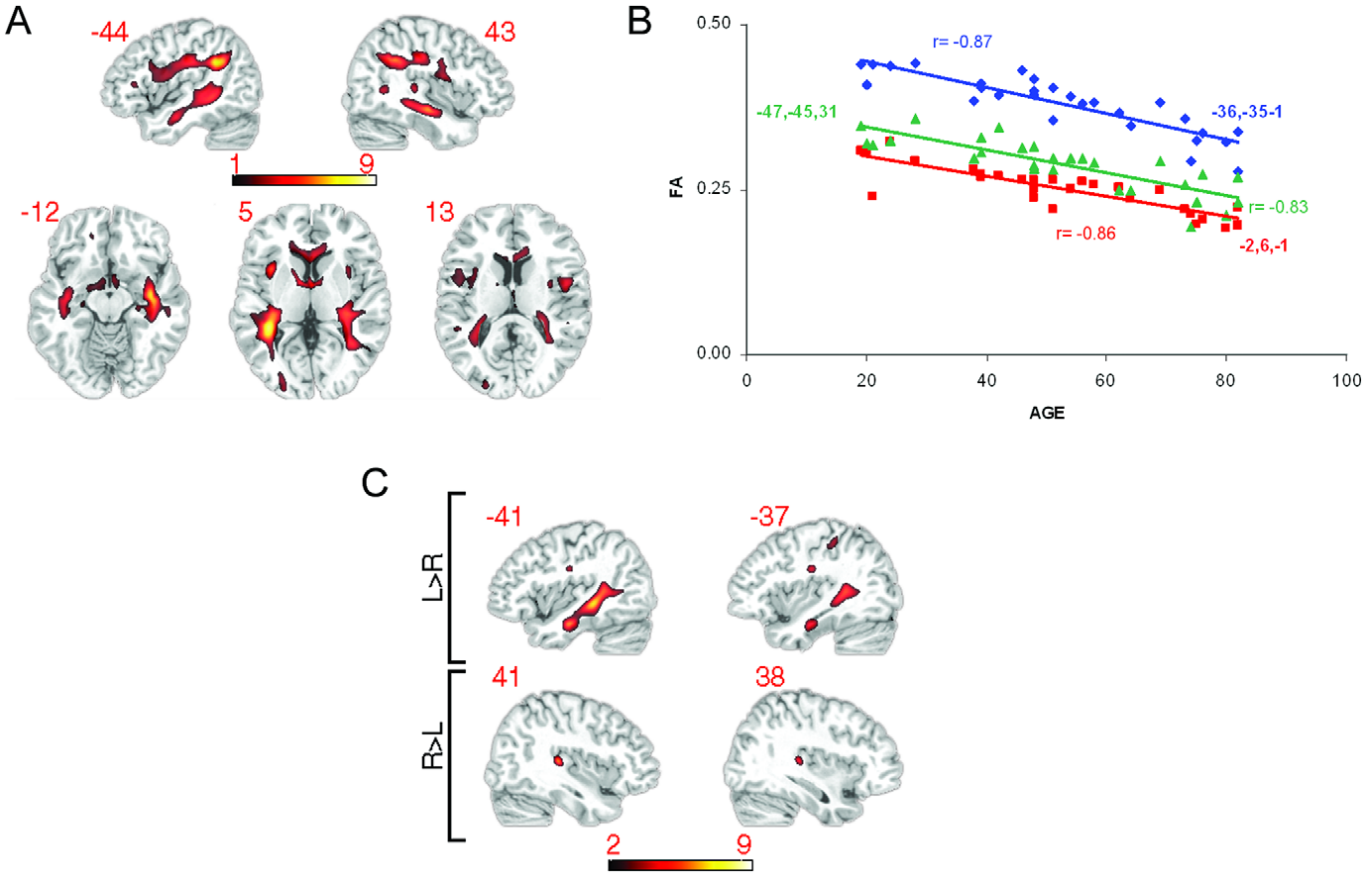
Fractional Anisotropy by age and hemisphere: a) Age-related reductions in FA are shown superimposed on a T1-weighted spatially normalized brain scan. Left side of the axial slices corresponds to left hemisphere. Color bar indicates range of t-scores. b) FA values obtained from all participants are shown plotted as a function of age. Mean FA values were obtained from 10 mm diameter spheres centered at the peaks of the three most significant voxels resulting from the whole brain SPM analysis. (i) −36 −35 −1 (*r* = −.87, *p*<.001), (ii) −47 −45 31 (*r* = −.83, *p*<.001) (iii) −2 6 −1 (*r* = −.86, *p*<.001). c) Cross-hemispheric FA comparisons are shown superimposed on a T1-weighted spatially normalized brain scan. Color bar indicates range of t-scores, with higher values reflecting greater asymmetry.

**Figure 3:**
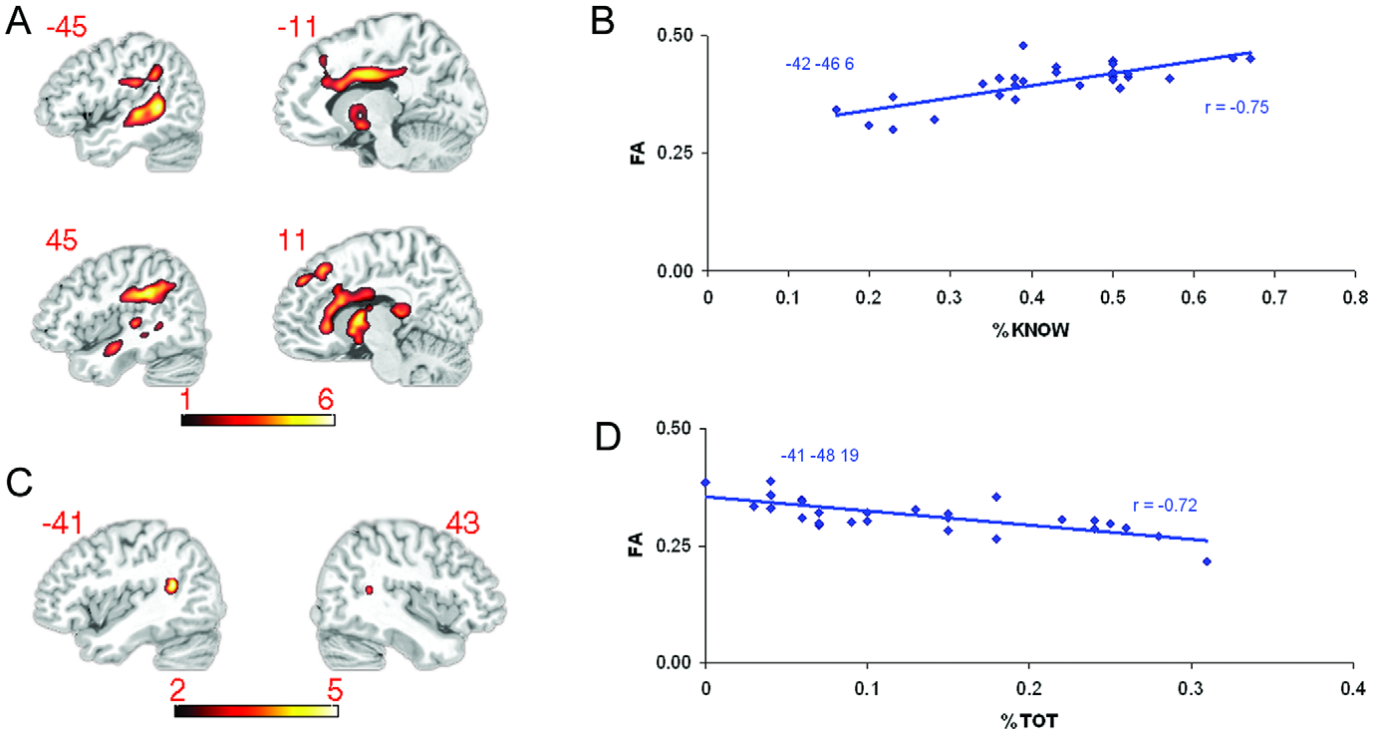
Fractional anisotropy and performance in the TOT task. a) Correlations between proportion Know responses from the TOT task and FA are shown superimposed on a T1-weighted spatially normalized brain scan. Color bar indicates range of t-scores. b) Mean FA values for each participant, obtained at the statistical peak of −42 −46 6 are shown plotted against proportion Know responses (*r* = −.75, *p*<.001). Mean FA values from each participant were obtained from a 10 mm diameter sphere centered at the peak of the statistically significant cluster. c) FA correlations between proportion TOTs and FA are shown superimposed on a T1-weighted spatially normalized brain scan. Color bar indicates range of t-scores. d) Mean FA values for each participant, obtained at the statistical peak of 41 −48 19 are shown plotted against proportion TOTs (*r* = −.72, *p*<.001). Mean FA values from each participant were obtained from a 10 mm diameter sphere centered at the peak of the statistically significant cluster.

Post-hoc analyses to confirm the voxel-wise findings were carried out and involved extracting mean FA values for spherical regions with 10 voxels diameter, which were centered at statistical peaks found in the voxel wise analysis. Although we would usually extract mean values for the entire significant cluster so that the effects sizes we report are representative of the whole cluster, in this instance we decided to use 10 mm diameter spheres because some of the significant clusters were very large (e.g. the cluster for the correlation of FA with age was 227611 voxels, spanning through most of the brain). The FA values extracted were correlated with variables of interest (e.g., age or TOT proportion). Pearson correlation coefficients and plots of the relationships between FA and variables of interest are shown in Figures 2 and 3. The FA data extracted for the plots in figure 3 originated from the set of analyses that did not include age as an additional variable. We used the JHU tractography atlas tool as implemented in FSLVIEW part of FSL (http://www.fmrib.ox.ac.uk/fsl/) to confirm major WM bundle identification and the Harvard-Oxford Cortical Structural Atlas as implemented on FSLVIEW to provide regional WM labels for the tables.

## Results

### 1. Behavioral results

Scores on the Shipley vocabulary test [25] increased with age, *r* = .62, *p*<.001 (Figure 1a), as did scores on the NART, *r* = .60, *p*<.001 (Figure 1b). The NART performance indicated that older adults made fewer pronunciation errors than younger adults, probably because older adults have larger vocabularies which would aid them in knowing how to pronounce the low frequency irregularly-spelled words which comprise the NART. Supporting this, the correlation of NART errors and age was no longer significant when Shipley scores were partialled out. Age was not correlated with digit spans forward or backward, or with score on the BNT.

Age was positively correlated with number of TOTs, *r* = .44, *p*<.01 (Figure 1c), negatively correlated with the proportion of “Know” responses, *r* = −.64, *p*<.001 (Figure 1d), and positively correlated with “Don't Know” responses, *r* = .47, *p*<.05 (Figure 1e). Additionally, TOT rates did not correlate significantly with measures of vocabulary knowledge, as measured by the Shipley vocabulary test. Know rates correlated negatively with Shipley scores, *r* = −.39, *p*<.05 (Figure 1f). However, this correlation did not remain significant when age was partialled out, indicating that the relationship was being mediated by the negative correlation between age and Know rates.

### 2. Fractional Anisotropy by age and hemisphere

Consistent with previous research, we found evidence for wide-spread age-related alteration in white matter tracts, which we identified by correlating FA scores with age in each voxel across the brain (see Figure 2a, 2b). This bilateral network included SLF, inferior longitudinal fasciculus, most of the fronto-occipital fasciculus, much of the cingulum and parts of the corpus callosum (rostrum, genu and body of the corpus callosum). In agreement with earlier studies [33], [34], posterior aspects of the corpus callosum including the splenium did not change significantly with age. Age-related reductions in FA were also observed at the anterior aspect of the left uncinate fasciculus, the anterior limb of the internal capsule, the fornix, tapetum and the posterior region of the corona radiata bilaterally (see Figure 2a). Taken together, these findings are largely in agreement with studies reporting white matter areas sensitive to age [35], [36], [33], [37], [38], [39]. Figure 2b confirms a linear decrease in FA over the sampled age range, showing the individual subject mean FA values plotted at the peak significant voxels for the three significant clusters.

Figure 2a shows bilateral regions of negative correlation between age and FA (also see Table S1), with higher t-scores in the left than right hemisphere, however this is a numerical difference that needs robust statistical assessment. Previous research indicates that a number of regions show higher FA values in the left hemisphere potentially signifying more white matter organization [31], [40], [41]. Because the broader theme of the paper is age-related language change and given findings indicating FA asymmetry primarily in the temporal lobe [31], [40], [41], an area of great importance to language function, it is essential to establish whether similar asymmetries exist in the current data set and whether these asymmetries are affected by age. We tested for this asymmetry by flipping the FA images along the x-axis for each participant and comparing the original spatially normalized and smoothed images to their flipped versions across the group. We made sure we compared homologous regions by spatially normalizing the images to a symmetrical template (see Methods). We found a number of regions with higher FA values in the left hemisphere when compared to the right hemisphere (see Figure 2c, Table S2). These regions were primarily in the temporal cortex, with the most significant left vs. right asymmetries in the inferior longitudinal fasciculus, the superior longitudinal fasciculus and the superior and anterior regions of corona radiata. There was only one area of right vs. left asymmetry located below the cortical ribbon in Heschel's gyrus (see Figure 2c). The presence of the predominately L>R asymmetry indicates our data are in keeping with previous findings. We then tested whether these hemispheric asymmetries were affected by age by obtaining a L - R difference image for each participant and correlating these difference images, at a voxel level, with age. Each voxel in the difference images indicates the magnitude of L vs. R difference. This correlation did not yield any significant results, suggesting that hemispheric asymmetries remain stable over the lifespan.

### 3. Fractional anisotropy and performance

Our main research question was whether age-related changes in white matter integrity are associated with age-related increases in rates of word retrieval failures. To address this question we first correlated successful retrieval (Know responses) rates in the TOT task and FA values across the brain. Proportion Know responses positively correlated with FA in the SLF, inferior longitudinal fasciculus, and the occipito-frontal fasciculi bilaterally (see Figure 3 and Table S3). Proportion Know responses also correlated extensively with the FA in the genu and the anterior aspect of the body of the corpus callosum bilaterally, and the middle aspect of the cingulum bilaterally. We also found significant correlations with proportion Know in posterior limb of the internal capsule, and the corticospinal and corticobulbar tracts. There were no significant negative correlations between FA and Know rates. In sum, increased coherence in the white matter bundles detailed above was associated with increases in the likelihood of participants correctly identifying the name of the target person.

Although correlations of FA with Know rates were widespread, TOT rates were negatively correlated with a FA only in the most posterior extent of the left SLF (located behind the most posterior aspect of the superior temporal gyrus; see Figure 3c and 3d and Table S4) and in a homologous area in the right hemisphere. There were no white matter structures that correlated positively with proportion TOTs. The findings from both the correlation with proportion Know responses and proportion TOT responses highlight the importance of an intact peri-Sylvian white matter network for word production. In both cases better retrieval - decreased TOTs or increased Knows - is related to higher FA values. The white matter regions that correlated with proportion TOTs were limited to the posterior aspect of the SLF and are a subset of the more extensive network that correlated with proportion Knows.

Although we expected age to be the primary determinant of variation in both FA and TOT rates, if the SLF is crucial to word production its integrity should be important across the age range. To test for this possibility, we carried out a whole brain multiple linear regression analysis in SPM using both age and proportion TOTs as predictor variables in the same model. The question we addressed with this analysis was whether proportion TOTs still uniquely predicts FA variance when age is accounted for. The clusters we report which correlate with TOT rates in the SLF remain significant in the multiple linear regression model, although t-scores for the peak voxels are reduced. For the left hemisphere cluster we found a peak t-score of 5.72 (*p*<.001) in the initial analysis that does not include age in the model, and a t-score of 4.63 (*p*<.001) when we included age in the multiple linear regression model. For the right hemisphere cluster we found a peak t-score of 5.63 (*p*<.001) when age was not included in the model and a t-score of 4.49 (*p*<.001) when age was included the multiple linear regression model.

Additional evidence that the relationship between FA and TOTs cannot be fully explained by a global aging effect comes from carrying out two voxel based whole brain correlations of Shipley vocabulary scores and NART scores with FA. Both of these behavioral measures significantly increased with age (Shipley vocabulary scores, r = .62, p<.001 and NART scores r = .60, p<.001); however neither of them correlated with FA anywhere in the brain. While it would be unexpected if increasing vocabulary scores was related to decreasing FA, these null results confirm that the relation of FA and proportion Know and TOT responses does not simply represent the relation of any behavioral score and age.

## Discussion

In this study we employed voxel-based analyses of FA images to establish age-related changes in WM integrity using DTI. The prevailing view of age–related WM changes is that ischemic events related to hypertension [42] cause these changes but decreases in myelinated axons may also play a role [43]. Comparable DTI studies [44] reported extensive FA decline with age and in keeping with those findings we observed a widespread pattern of age-related FA decreases that were not uniform throughout the brain. Fronto-temporal FA decreases were more prominent in our data with FA in occipital areas appearing relatively spared from the aging process, suggesting an anteroposterior gradient in the effect of aging. This anterior-to-posterior age-related gradient of change, especially in the corpus callosum, has been previously reported in both region of interest (ROI) and voxel based analyses [35], [36], [33], [37], [38], [39], and has also been established with relaxometry measures [45]. This pattern of change was reversed in periventricular areas. We found an opposite gradient of decline (in agreement with Salat et al., 2005 [33]) with posterior periventricular regions showing a greater decline in FA than anterior ones (see Figure 2a). Although periventricular abnormalities are often noted in aging, the specific pattern we observed here has rarely been reported [33].

Given the fact that even the best spatial normalization procedure will not equate the ventricles of the older participants to those of the youngest participants there remains the possibility that the periventricular effect we report may be an artifact caused by spatial normalization or even smoothing. However, we confirmed with visual inspection that both smoothing and spatial normalization had a similar effect in anterior and posterior aspects of periventricular brain regions and therefore normalization or smoothing are unlikely explanations for the greater posterior periventricular age-related changes we found.

Another question we attempted to answer with this study was whether hemispheric FA asymmetries exist and whether they change with age. We replicated previous findings of hemispheric asymmetries in FA measures, demonstrating predominantly left temporal lobe advantage, which may signify increased organization in WM tissue supporting language function [31], [46], [16]. Our relatively novel finding is that these asymmetries persist over the lifespan. Given that there are fMRI studies proposing older individuals recruit increasingly more bilaterally distributed brain regions compared to younger adults in response to the same task, [47] our finding of stable hemispheric asymmetry is somewhat unexpected (but see [48]). If older individuals recruit additional contralateral regions as suggested by fMRI, the expectation would be that contralateral WM organization increases to support these activations. Such WM tissue changes would cause a decrease in left/right FA asymmetry. Contrary to this our findings suggest that left/right WM asymmetries remain stable throughout the lifespan. One potential explanation is that despite the global reduction FA there is enough residual WM integrity to support the age-related bilateral activation patterns.

Finally, we turn our attention to the main question we set out to answer with this study: whether age-related changes in white matter integrity are associated with age-related increases in rates of word retrieval failures. Word-finding failures constitute a major complaint in old age [11] and by using a picture naming task we found that the proportion of correct naming (Know) responses correlated with FA in a number of WM bundles. These included primarily the SLF, inferior longitudinal fasciculus, cingulum, and also the corpus callosum, i.e. an extensive white matter network that is necessary for successful word production. These white matter tracts connect cortical regions known to be involved in language production, principally the posterior extent of the superior and middle temporal gyri to the inferior frontal cortex. Although the integrity of a wide range of tracts was related to Know responses, proportion TOTs correlated with FA only in a subset of these tracts. Specifically, TOT rates correlated negatively with FA in the most posterior aspect of the SLF at the level of the posterior superior temporal gyrus. Hence, age-related increases in TOTs appear to be related to age-related deterioration in the integrity of a specific white matter tract, which plays a major role in language function. The SLF arcs around the Sylvian fissure to connect frontal, posterior temporal and inferior parietal regions. Findings in this study, as well as others [31], [46], [16], propose that there is a leftward asymmetry in the SLF, suggesting the SLF plays a role in language function. The question remains which type of language function the SLF subserves.

Wernicke, 1874 [49] claimed that the SLF was the most relevant WM tract for language function and that disruption to the SLF leads to frontotemporal disconnection resulting in conduction aphasia, a type of aphasia characterized by poor speech repetition. This model lacked anatomical specificity and implicated all of the SLF in language production and specifically phonological processing. Data from more recent DTI studies indicates that only the superior temporal gyrus terminations of the SLF are implicated in phonological processing [20]. Additionally, the Wernicke WM model has been disputed by studies showing that conduction aphasia is possible with lesions in insular grey matter [50] an area shown in our previous research [15], [51] to play a role in age-related phonological retrieval. Thus, it may be that damage to areas involved in the phonological network, whether grey matter (such the insula) or white matter (such as the SLF), can result in impaired phonological processing. This is supported by previously-reported functional and structural data published by our group [15], [51] and by the current findings.

Additional evidence for the role of SLF in phonological processing comes from research implicating SLF abnormalities in the phonological impairments seen in developmental dyslexia [52]. Many previous researchers have suggested functional separation of different segments of the SLF [16], so it may be that the posterior aspect (where we find a correlation between FA and TOTs) has particular significance for phonological retrieval. Another possibility is that a more extensive region of SLF is relevant for phonological retrieval, but FA in the most posterior aspect correlated with age-related increases in word-finding failures because this region was particularly vulnerable to the effect of age (see Figure 2). The aspect of the SLF we found to correlate with TOTs, behind the posterior extent of the superior temporal gyrus, not only further substantiates the notion that the SLF is involved in phonological retrieval but also strengthens our belief that TOTs in old age may result from a temporary inability to access the phonology of a word, following successful activation of semantic information [53], [13].

With the data we present here we do not propose that the SLF is dealing with phonological processing alone. There has been a plethora of recent DTI studies providing evidence for a more complex system of tracts linking posterior temporoparietal and frontal cortical regions, most of which are likely to be involved in multiple aspects of language function [18], [54], [19].

Although our previous [15] and current findings demonstrate the potential for integrating the role of insula grey matter and SLF white matter connections in word production failures, there are notable differences in the correlations between performance and white matter in the current study and grey matter in our previous research [15]. For example, whereas in the current study WM correlated with both Know and TOT scores, in our previous research, grey matter correlated with TOT rates [15], but demonstrated no significant correlation with Know rates anywhere in the brain (unreported result from the same dataset). The implication is that although grey and white matter may be integrated into the same functional network, their sensitivity to the modulation of specific processes involved in word production differs.

One general point that needs to be made is that despite the fact that FA images contain information on grey matter fractional anisotropy none of the analyses we report here produced significant findings in grey matter areas. This suggests that FA is not a sensitive enough measure of age-related changes in grey matter probably because directionality of diffusion in grey matter does not change significantly with age. GM density, as derived from T1 weighted images, is a more sensitive measure of GM related changes as we have reported in Shafto et al., 2007 [15].

Finally, although we believe our method provides the best chance of identifying the relationship between FA change and performance in old age, some criticism has been directed at a voxel-based approach to FA analysis [55], [56]. An alternative method, tract-based spatial statistics [57] aims to reduce the error introduced by imperfect tract registration by assessing only the centers of major tracts. In this paper, these registration issues were addressed by careful inspection of the images and adaptation of our methods in order to allow assessment of the whole tract volume including tract periphery. We also note that several of our findings replicate findings from other groups that have used alternative WM analysis methods. For example, using our voxel-based approach we were able to replicate an anteroposterior gradient in the way aging affects WM. This specific gradient of change has been previously reported in both region of interest (ROI) and voxel based analyses [35], [36], [33], [37], [38], [39] Moreover, we replicated the previous finding of hemispheric asymmetries in FA measures, demonstrating predominantly left temporal lobe advantage [31], [46], [16], [48].

In conclusion, our findings indicate that age-related increases in word finding failures represent a specific rather than universal cognitive decline, corroborating previous behavioral data [12], [13] as well as evidence from grey matter structure [15] and fMRI [51]. We found widespread changes in FA with age, and positive correlation of FA and successful retrieval rates throughout the WM structures supporting a cortical grey matter language network. However, we found a specific correlation between FA decline and increasing TOT rates in SLF, a region important for language production. Finally, the current findings in combination with previous evidence from grey matter measures demonstrate the importance of examining both grey and white matter when characterizing brain-behavior relationships in old age.

## Supporting Information

Table S1 Statistical peaks resulting from the correlation of age and FA.
(0.03 MB DOC)

Table S2 Statistical peaks resulting from the comparison of LH vs. RH FA.
(0.07 MB DOC)

Table S3 Statistical peaks resulting from the correlation of FA and % Knows.
(0.04 MB DOC)

Table S4 Statistical peaks resulting from the correlation of FA and %TOTs.
(0.03 MB DOC)
