# Supplementary material for: White Matter Changes and Word Finding Failures with Increasing Age
Source: PLoS One. 2011 Jan 7;6(1):e14496. doi: 10.1371/journal.pone.0014496 (PMC3017545; doi:10.1371/journal.pone.0014496)
Supplement: Table S1 — Statistical peaks resulting from the correlation of age and FA. (0.03 MB DOC) [file pone.0014496.s001.doc]

**Table S1.** Statistical peaks resulting from the correlation of age and FA.

| Cluster (p)  corrected | Voxel (p)  corrected | Voxel  T | Voxel  Equiv. Z | Voxel (p)  Uncorrected | x,y,z (mm) | WM tracts/regional labels |
| --- | --- | --- | --- | --- | --- | --- |
| <0.001 | <0.001 | 10.70 | 6.57 | <0.001 | -36,-35,-1 | L Retrolenticular part of internal capsule L |
|  | <0.001 | 9.62 | 6.23 | <0.001 | -47,-45,31 | L Supramarginal Gyrus WM |
|  | <0.001 | 9.20 | 6.09 | <0.001 | -2 6 -1 | L Anterior limb of internal capsule |
