# Supplementary material for: White Matter Changes and Word Finding Failures with Increasing Age
Source: PLoS One. 2011 Jan 7;6(1):e14496. doi: 10.1371/journal.pone.0014496 (PMC3017545; doi:10.1371/journal.pone.0014496)
Supplement: Table S2 — Statistical peaks resulting from the comparison of LH vs. RH FA. (0.07 MB DOC) [file pone.0014496.s002.doc]

**Table S2.** Statistical peaks resulting from the comparison of LH vs. RH FA.

| Cluster (p)  corrected | Voxel (p)  Corrected | Voxel  T | Voxel  Equiv. Z | Voxel (p)  Uncorrected | x,y,z (mm) |  |
| --- | --- | --- | --- | --- | --- | --- |
| <0.001 | <0.001 | 11.08 | 6.68 | <0.001 | -47,-10,-19 | L Middle Temporal Gyrus WM |
|  | <0.001 | 10.15 | 6.4 | <0.001 | -46,-31,-3 | L Middle Temporal Gyrus WM |
|  | <0.001 | 8.02 | 5.64 | <0.001 | -53,-15,-24 | L Inferior Temporal Gyrus WM |
| <0.001 | <0.001 | 9.85 | 6.3 | <0.001 | -27,-28,52 | L Precentral Gyrus WM |
|  | 0.015 | 3.63 | 3.23 | 0.001 | -17,-30,57 | L Precentral Gyrus WM |
|  | 0.016 | 3.60 | 3.21 | 0.001 | -32,-37,57 | L Postcentral Gyrus WM |
| <0.001 | <0.001 | 8.68 | 5.9 | <0.001 | 9,52,-18 | R Frontal Pole WM |
|  | 0.014 | 3.66 | 3.25 | 0.001 | 5,37,-14 | R Frontal Medial Cortex WM |
| <0.001 | <0.001 | 8.47 | 5.82 | <0.001 | 41,-28,10 | R Retrolenticular part of Internal Capsule WM |
|  | <0.001 | 6.80 | 5.11 | <0.001 | 57,-36,19 | R Planum Temporale WM |
|  | 0.001 | 5.13 | 4.22 | <0.001 | 58,-27,11 | R Planum Temporale WM |
| <0.001 | <0.001 | 8.12 | 5.68 | <0.001 | -15,-57,16 | L Splenium of corpus callosum |
|  | 0.003 | 4.52 | 3.85 | <0.001 | -12,-67,28 | L Precuneous WM |
| <0.001 | <0.001 | 6.58 | 5.01 | <0.001 | -30,3,29 | L Superior longitudinal fasciculus |
|  | <0.001 | 5.65 | 4.52 | <0.001 | -22,-3,25 | L Superior corona radiata |
|  | 0.001 | 5.12 | 4.22 | <0.001 | -38,-13,28 | L Superior longitudinal fasciculus L |
| <0.001 | <0.001 | 6.41 | 4.92 | <0.001 | 16,-65,5 | R Intracalcarine Cortex WM |
|  | 0.003 | 4.55 | 3.87 | <0.001 | 10,-67,10 | R Intracalcarine Cortex WM |
| <0.001 | 0.001 | 5.30 | 4.33 | <0.001 | 32,9,-17 | R Uncinate fasciculus |
|  | 0.001 | 4.93 | 4.11 | <0.001 | 27,21,-13 | R Frontal Orbital Cortex WM |
|  | 0.001 | 4.92 | 4.10 | <0.001 | 30,28,-1 | R Frontal Orbital Cortex WM |
| <0.001 | 0.001 | 5.21 | 4.27 | <0.001 | 32,-44,34 | R Superior longitudinal fasciculus |
|  | 0.001 | 4.97 | 4.13 | <0.001 | 16,-45,28 | R Splenium of corpus callosum |
|  | 0.002 | 4.71 | 3.97 | <0.001 | 22,-45,21 | R Splenium of corpus callosum |
| <0.001 | 0.001 | 5.19 | 4.26 | <0.001 | -4,41,0 | L Paracingulate Gyrus WM |
|  | 0.001 | 5.07 | 4.19 | <0.001 | -4,36,7 | L Genu of corpus callosum |
|  | 0.002 | 4.65 | 3.93 | <0.001 | -7,20,20 | L Genu of corpus callosum |
| <0.001 | 0.001 | 5.03 | 4.17 | <0.001 | -44,-25,20 | L Parietal Operculum WM |
| <0.001 | 0.001 | 4.96 | 4.12 | <0.001 | -60,-22,22 | L Postcentral Gyrus WM |
