# Supplementary material for: White Matter Changes and Word Finding Failures with Increasing Age
Source: PLoS One. 2011 Jan 7;6(1):e14496. doi: 10.1371/journal.pone.0014496 (PMC3017545; doi:10.1371/journal.pone.0014496)
Supplement: Table S3 — Statistical peaks resulting from the correlation of FA and % Knows. (0.04 MB DOC) [file pone.0014496.s003.doc]

**Table S3.** Statistical peaks resulting from the correlation of FA and % Knows.

| Cluster (p)  corrected | Voxel (p)  corrected | Voxel  T | Voxel  Equiv. Z | Voxel (p)  Uncorrected | x,y,z (mm) |  |
| --- | --- | --- | --- | --- | --- | --- |
| <0.001 | 0.001 | 7.37 | 5.37 | <0.001 | 11,7,0 | R Anterior limb of Internal Capsule |
|  | 0.001 | 6.56 | 5.00 | <0.001 | 25,-29,10 | R Retrolenticular part of internal capsule |
|  | 0.001 | 6.35 | 4.89 | <0.001 | 22,-27,-13 | R Cingulum (hippocampus) |
| <0.001 | 0.001 | 6.76 | 5.09 | <0.001 | -42,-46,6 | L Superior longitudinal fasciculus L |
|  | 0.001 | 6.37 | 4.90 | <0.001 | -24,-26,15 | L Retrolenticular part of internal capsule |
|  | 0.001 | 5.35 | 4.35 | <0.001 | -49,-29,2 | L Superior Temporal Gyrus WM |
| 0.007 | 0.001 | 5.61 | 4.50 | <0.001 | -31,-73,28 | L Lateral Occipital Cortex WM |
|  | 0.002 | 4.98 | 4.14 | <0.001 | -21,-68,27 | L Cuneus WM |
| 0.011 | 0.001 | 5.33 | 4.34 | <0.001 | -18,-30,47 | L Precentral Gyrus WM |
|  | 0.004 | 4.37 | 3.75 | <0.001 | -22,-22,47 | L Superior corona radiata |
| <0.001 | 0.002 | 4.82 | 4.04 | <0.001 | -50,-45,36 | L Supramarginal Gyrus WM |
|  | 0.004 | 4.36 | 3.74 | <0.001 | -47,-29,26 | L Parietal Operculum WM |
|  | 0.005 | 4.14 | 3.60 | <0.001 | -38,-43,35 | L Supramarginal Gyrus WM |
