# Supplementary material for: White Matter Changes and Word Finding Failures with Increasing Age
Source: PLoS One. 2011 Jan 7;6(1):e14496. doi: 10.1371/journal.pone.0014496 (PMC3017545; doi:10.1371/journal.pone.0014496)
Supplement: Table S4 — Statistical peaks resulting from the correlation of FA and %TOTs. (0.03 MB DOC) [file pone.0014496.s004.doc]

**Table S4.** Statistical peaks resulting from the correlation of FA and %TOTs.

| Cluster (p)  corrected | Voxel (p)  corrected | Voxel  T | Voxel  Equiv. Z | Voxel (p)  Uncorrected | x,y,z (mm) |  |
| --- | --- | --- | --- | --- | --- | --- |
| 0.003 | 0.027 | 5.72 | 4.56 | <0.001 | -41,-48,19 | L Superior Longitudinal Fasciculus |
|  | 0.067 | 3.67 | 3.27 | 0.001 | -62,-59,14 | L Angular Gyrus WM |
|  | 0.027 | 5.63 | 4.51 | <0.001 | 55,-49,17 | R Angular Gyrus WM |
| <0.001 | 0.042 | 4.35 | 3.74 | <0.001 | 61,-30,16 | R Superior temporal gyrus WM |
|  | 0.051 | 4.13 | 3.59 | <0.001 | 43,-47,14 | R Superior Longitudinal Fasciculus |
